# Efficient biosynthesis of β-caryophyllene by engineered *Yarrowia lipolytica*

**DOI:** 10.1186/s12934-025-02660-w

**Published:** 2025-02-06

**Authors:** Young-Kyoung Park, Lucie Studena, Piotr Hapeta, Ramdane Haddouche, David J. Bell, Pablo Torres-Montero, Jose Luis Martinez, Jean-Marc Nicaud, Adriana Botes, Rodrigo Ledesma-Amaro

**Affiliations:** 1 Department of Bioengineering and Imperial College Centre for Synthetic Biology, Imperial College London, London, UK; 2 Université Paris-Saclay, INRAE, AgroParisTech, Micalis Institute, Jouy-en-Josas, 78350 France; 3 VideraBio Ltd, Derbyshire, UK; 4 SynbiCITE Innovation and Knowledge Centre, Imperial College London, London, UK; 5 Department of Biotechnology and Biomedicine, Section for Synthetic Biology, Technical University of Denmark, Kongens Lyngby, Denmark; 6 Bezos Centre for Sustainable Protein, Imperial College London, London, UK; 7 UKRI Mission Hub on Microbial Food, Imperial College London, London, UK

**Keywords:** Β-caryophyllene, *Yarrowia Lipolytica*, Terpene, Bioproduction, Synthetic biology

## Abstract

**Background:**

β-Caryophyllene, a sesquiterpenoid, holds considerable potential in pharmaceutical, nutraceutical, cosmetic, and chemical industries. In order to overcome the limitation of β-caryophyllene production by the extraction from plants or chemical synthesis, we aimed the microbial production of β-caryophyllene in non-conventional yeast *Yarrowia lipolytica* in this study.

**Results:**

Two genes, *tHMG1* from *S. cerevisiae* to boost the mevalonate pool and *QHS1* from *Artemisia annua*, were expressed under different promoters and copy numbers in *Y. lipolytica*. The co-expression of 8UAS pEYK1-*QHS1* and pTEF-*tHMG1* in the obese strain yielded 165.4 mg/L and 201.5 mg/L of β-caryophyllene in single and double copies, respectively. Employing the same combination of promoters and genes in wild-type-based strain with two copies resulted in a 1.36-fold increase in β-caryophyllene. The introduction of an additional three copies of 8UAS pEYK1-*tHMG1* further augmented the β-caryophyllene, reaching 318.5 mg/L in flask fermentation. To maximize the production titer, we optimized the carbon source ratio between glucose and erythritol as well as fermentation condition that led to 798.1 mg/L of β-caryophyllene.

**Conclusions:**

A biosynthetic pathway of β-caryophyllene was firstly investigated in *Y. lipolytica* in this study. Through the modulation of key enzyme expression, we successfully demonstrated an improvement in β-caryophyllene production. This strategy suggests its potential extension to studies involving the microbial production of various industrially relevant terpenes.

**Supplementary Information:**

The online version contains supplementary material available at 10.1186/s12934-025-02660-w.

## Introduction

Terpenoids, secondary metabolites widely found in plants, have great applications in food, pharmaceutical, cosmetics, biofuels, and so on [1]. Their biological functions and applications are diverse depending on molecular architectures categorized into mono- (C10), sesqui- (C15), di- (C20), tri- (C30) and tetraterpenoids (C40). The natural sources of terpenoids are limited to the plants that cause low yield, high production cost, shortage of natural resources, and so on. Microbial production of terpenoids by synthetic biology has gained immense interest from researchers as a promising alternative to traditional terpenoid productions [1].

β-Caryophyllene, one of the most abundant caryophyllenes among α, β, and γ types in nature, is a plant sesquiterpenoid which has potential in various industries. It has been regarded as a potential therapeutic compound due to its antioxidant, anti-inflammatory, and anticarcinogenic properties [2, 3]. It also has applications as fragrances, precursors of flavor, and potential aircraft fuels [4, 5]. Conventional production of this compound is still limited to chemical synthesis or extraction from plant tissue that shows the challenges by low production titers, insufficient recovery yields, environmental pollution, and high cost attributed to the intricate extraction process [6, 7]. Therefore, the microbial production of β-caryophyllene is gaining an interest as a promising alternative to conventional production by its feasibility and sustainability of the production process.

Microbial production of β-caryophyllene is quite limited so far. The first report of β-caryophyllene production was described by expressing β-caryophyllene synthase (QHS1 from *Artemisia annua*) in *E. coli* [8]. The authors used acetic acid as a feedstock and engineered the strain by overexpressing acetyl-CoA synthase (ACS), acetoacetyl-CoA synthase (AACS) to increase precursor pools of β-caryophyllene from acetic acid. For further increase of β-caryophyllene synthesis, hybrid mevalonate pathway was overexpressed in the engineered strain resulting in 1.05 g/L of β-caryophyllene from acetic acid in batch fermentation. Another study screened several origins of QHS1 in *E. coli*, the strain harboring QHS1 from *Artemisia annua* was selected for further engineering [8]. Using a multi-step metabolic engineering strategy designed to increase precursor (GPPS2 from *Abies grandis*) and supply NADPH cofactor (native G6PDH) for β-caryophyllene production, the final engineered strain produced 1.52 g/L of β-caryophyllene during 60 h after induction in aerobic fed-batch fermentation with glucose.

In *Saccharomyces cerevisiae*, the production of β-caryophyllene was increased by adaptive laboratory evolution in oxidative stress [9]. The best evolved mutant (STE6, ABC transporter) achieved a titer of 104.7 mg/L of β-caryophyllene by likely increasing product export. Another group developed *S. cerevisiae* as a sesquiterpene chassis strain by enhancing endogenous mevalonate pathway [10]. The overexpression of *QHS1* from *Artemisia annua* in the chassis strain resulted in 250.4 mg/L and 2949.1 mg/L of β-caryophyllene production in shake flask and fed-batch fermentation, respectively (Supplementary Table 1) [10].

*Yarrowia lipolytica*, one of well-known non-conventional yeasts, is often regarded as a promising chassis in synthetic biology and industrial biotechnology. Its safety, genetical stability, efficient synthetic biological tools, and ability to utilize various carbon sources make this yeast a good host for bioproduction [11–14]. The high flux toward acetyl-CoA, inherent mevalonate pathway, and naturally hydrophobic microenvironments of this yeast have been great advantages for synthesizing and accumulating terpenoids compared to other hosts [15–17]. Thus, various terpenoids including mono-, tri-, tetra- and sesquiterpenoids have already been produced in *Y. lipolytica* with diverse metabolic engineering strategies such as increasing precursor or cofactor supply, rewiring the mevalonate pathway, regulating lipid metabolism, and engineering catalytic efficiency of terpenoid synthases individually or iteratively [16, 17].

In this study, we engineered the yeast *Y. lipolytica* for producing β-caryophyllene through metabolic engineering and optimization of substrates. The β-caryophyllene synthase was heterologously expressed in different genetic backgrounds to evaluate their effect on the production and assess *Y. lipolytica* as a potential platform for terpenoid production. Also, substrate combination including glucose and erythritol was optimized for higher production of β-caryophyllene.

## Materials and methods

### Media and strains

Media and growth conditions for *E. coli* were previously described by Sambrook and Russell [18]; those for *Y. lipolytica* were previously described by Barth and Gaillardin [19].

Rich medium (YPD) was prepared containing 1% (w/v) yeast extract, 2% (w/v) peptone, and 2% (w/v) glucose. Minimal glucose medium (YNBD) was prepared containing 0.17% (w/v) yeast nitrogen base (without amino acids and ammonium sulphate, YNBww), 0.5% (w/v) NH_4_Cl, 50 mM KH_2_PO_4_–Na_2_HPO_4_ (pH 6.8), and 2% (w/v) glucose. To complement strain auxotrophy, 0.1 g/L of uracil or leucine was added as necessary. Solid media were prepared by adding 2% (w/v) agar.

Construction of plasmids were carried out by restriction enzyme digestion and ligation. Two restriction enzymes, BamHI and AvrII were used for cloning into JMP62-based plasmids. For cloning the gene into dual expression plasmids (DCU and DCL), different sets of restriction enzymes were used depending on promoters (HindIII and SalI under 8UAS EYK; BamHI and AvrII under pTEF) (Supplementary Table 2). Heterologous genes were synthesized and codon optimized to *Y. lipolytica* (Supplementary Table 2).

To introduce gene expression cassette into *Y. lipolytica*, the plasmids were first linearized using NotI and then transformed into competent cells using the lithium acetate/DTT method. The gene expression cassettes were randomly integrated into the genome of *Y. lipolytica*. Transformants were selected on YNBD media containing the appropriate amino acids for their specific genotype. Positive transformants were then confirmed by colony PCR with Phire Plant Direct PCR master mix (Thermo Fisher, Waltham, USA). Primers used for colony PCR are listed in Supplementary Table 3. Multicopy integration was performed by recycling the selective marker via the Cre-LoxP system. Plasmids and strains used in this study are listed in Table 1.

**Table 1:** Plasmids and strains used in this study

Plasmid		Reference
VB.ECO.242	DCU-URA3 ex-pTEF-tHMG	This study
VB.ECO.244	DCL-LEU2 ex-pTEF-tHMG	This study
JME4230	JMP62-URA3 ex-8UAS-EYK	[23]
VB.ECO.246	JMP62-URA3 ex-8UAS-EYK-tHMG	This study
VB.ECO.248	JMP62-LEU2 ex-8UAS-EYK-tHMG	This study
VB.ECO.473	JMP62-URA3 ex-8UAS pEYK-QHS1-pTEF-tHMG	This study
VB.ECO.475	JMP62-LEU2 ex-8UAS pEYK-QHS1-pTEF-tHMG	This study
VB.ECO.244	JMP62-LEU2 ex-8UAS pEYK-tHMG	This study
*Yarrowia lipolytica*		
JMY195	Po1d MATa ura3-302 leu2-270 xpr2-322	[19]
JMY2900	Po1d MATa ura3-302 leu2-270 xpr2-322 URA3 LEU2	[28]
S1266	Po1d URA3 ex-8UAS pEYK-QHS1-pTEF-tHMG + LEU2 ex-8UAS pEYK-QHS1-pTEF-tHMG	This study
S610	Po1d (8UAS pEYK-tHMG) * 3 Copies + (8UAS pEYK-QHS1-pTEF-tHMG) * 2Copies	This study
S1059 (JMY3820)	Po1d Δ*pox1-6* Δ*tgl4* pTEF-DGA2 pTEF-GPD1 Δ*eyk1* URA3 LEU2	[28]
S1090	S1059 8UAS pEYK-QHS1-pTEF-tHMG + LEU2	This study
S1093	S1059 8UAS pEYK-QHS1-pTEF-tHMG + LEU2 ex-8UAS pEYK-tHMG	This study
S1100	S1059 (8UAS pEYK-QHS1-pTEF-tHMG) * 2 copies	This study
S1106	S1059 (8UAS pEYK-QHS1-pTEF-tHMG) * 2 copies + LEU2 ex-8UAS pEYK-tHMG	This study

* Plasmids are written with an order: backbone plasmid-selective marker-promoter-expressed gene. The term ‘ex’ means excisable marker

### β-Caryophyllene production in flask

The strains were inoculated in YPD medium and cultivated overnight at 30 °C, 250 rpm. Cells were washed with distilled water and inoculated in YPDE (glucose and erythritol) medium with initial OD 0.1. Concentration of glucose and erythritol was variable depending on the strain which is indicated in each experiment. 20% (v/v) of dodecane was added to flask as organic extractant phase. The strains were grown at 30 °C, 250 rpm for 96 h. We used at least two biological replicates and calculated average and standard deviation values.

### Batch fermentation

The strains were inoculated in YPD medium and cultivated overnight at 30 °C at 250 rpm. Cells were washed with distilled water and inoculated into the 400 mL of YPD1E3 media in a 1 L bioreactor (Sartorius, Germany). 20% (v/v) of dodecane was added to the bioreactor as an organic extractant phase. The culture was carried out at 30 °C with a stirring speed of 200 rpm and an airflow rate of 1 vvm. pH was adjusted and maintained at 6.8 by adding 1 M HCl and 1 M NaOH.

The samples were taken during fermentation to measure the cell growth (OD and DCW), the concentration of substrates, and the concentration of caryophyllene. We used two biological replicates and calculated average and standard deviation values.

### Analysis

The cell growth was measured by the OD at 600 nm using a spectrophotometer (WPA Biowave II). The dry cell weight was obtained by drying biomass after washing it with distilled water.

The substrates (glucose, erythritol) used in this study were quantified by HPLC. Supernatants of the culture were analyzed by the UltiMate 3000 system (Thermo Fisher Scientific, UK) using an Aminex HPX-87 H column (300 mm x 7.8 mm, Bio-RAD, USA) coupled to UV and RI detectors. Glucose and erythritol were analyzed by the RI detector. The mobile phase used was 0.01 N H_2_SO_4_ with a flow rate of 0.6 mL/min and the column temperature was T = 30 °C. Identification and quantification were achieved via comparisons to standards.

β-Caryophyllene in the dodecane phase was quantified by GC-MS with a standard curve of (-)-trans-caryophyllene (GC grade, Sigma-Aldrich). After centrifuging the culture, the dodecane phase was directly injected into GC-MS. The following parameters were used for the samples: The substances were measured using the instrument Agilent 5977 A GC-MSD and column Agilent DB5-MS. The injection parameters were: 240 °C, a Split ratio of 20:1, and 1 µl of sample solution injected. The following temperature program was used: 100 °C for 2 min; 165 °C at 10 °C/min; 170 at 1.5 °C/min; 170 °C for 2 min; 187 °C for 1.5 °C/min; 280 at 30 °C/min; 280 °C for 2 min; Mass spectra: EI, positive, m/z 40–600, 1.3 spectra/sec. We used at least two biological replicates and calculated average and standard deviation values.

## Result

### Biosynthesis of β-caryophyllene

**Fig. 1:**
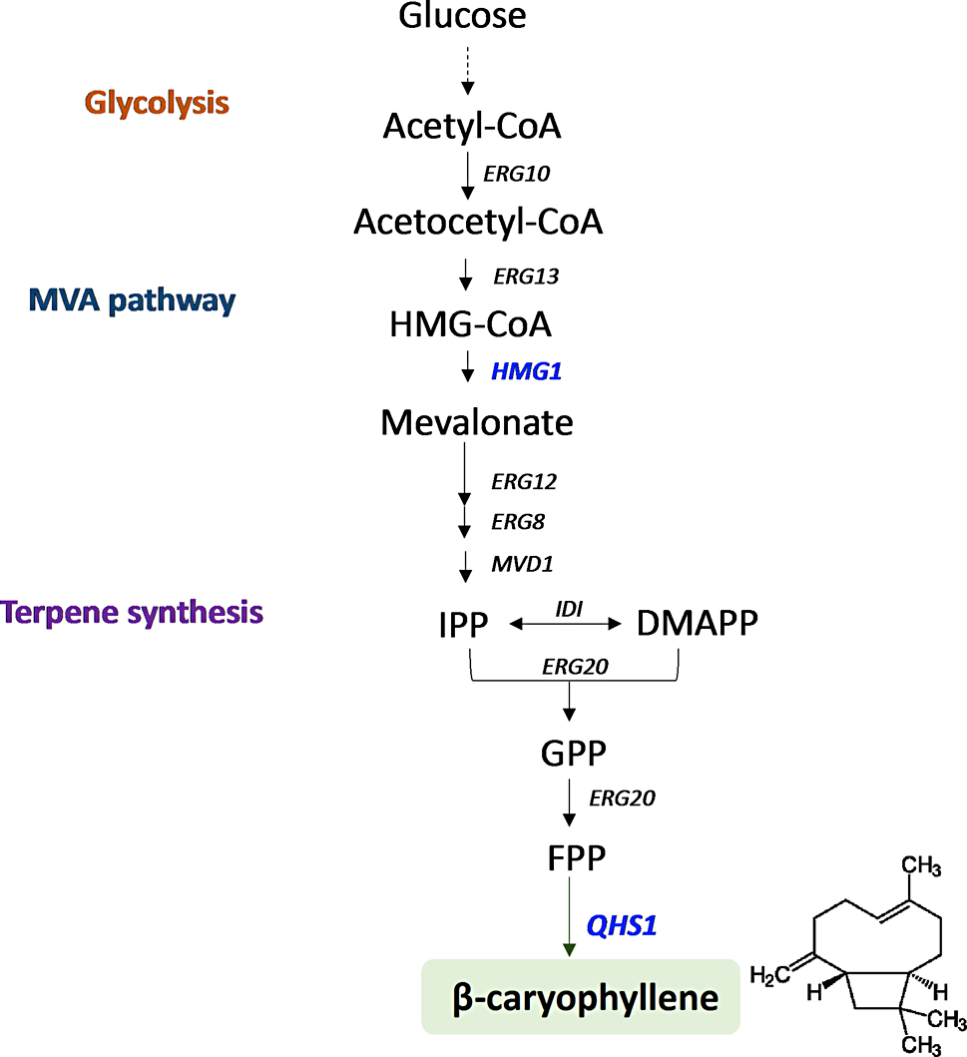
Biosynthetic pathway of β-caryophyllene. The genes overexpressed in this study are indicated in blue. Abbreviation; HMG-CoA, hydroxy-3-methylglutaryl-CoA; IPP, isopentenyl diphosphate; DMAPP, dimethylallyl diphosphate; GPP, geranyl diphosphate; FPP, farnesyl diphosphate; ERG10, acetoacetyl-CoA thiolase; ERG13, hydroxymethylglutaryl-CoA synthase; HMG1, HMG-CoA reductase; ERG12, mevalonate kinase; ERG8, phosphomevelaonate kinase; MVD1, mevalonate pyrophosphate decarboxylase; IDI, isopentenyl diphosphate isomerase; ERG20, farnesyl pryophosphoate synthetase; QHS1, caryophyllene synthase

The synthesis of β-caryophyllene is started from the mevalonate (MVA) pathway as other terpene synthesis in yeast. Mevalonate is converted to the common precursors of sesquiterpenes, farnesyl diphosphate (FPP), by going through isopentenyl diphosphate (IPP), dimethylallyl diphosphate (DMAPP), and geranyl diphosphate (GPP) as described in Fig. 1.

It is considered that the reaction from hydroxy-3-methylglutaryl-CoA (HMG-CoA) to mevalonate by HMG1 (HMG-CoA reductase) is the major rate-limiting step in terpenoid biosynthesis in many organisms [15, 16]. The truncated form of HMG1 (tHMG1) lacking the N-terminal domain has shown as an advantageous strategy for terpenoid production due to higher protein expression with a soluble form [16]. The heterologous overexpression of HMG1 was also shown effective for terpene production in *Y. lipolytica* by additionally boosting mevalonate pools apart from the native regulation in the native MVA pathway [20]. Therefore, we overexpressed *tHMG1* from *S. cerevisiae* to boost the mevalonate pool and the codon-optimized *QHS1* from *A. annua* for β-caryophyllene production in *Y. lipolytica* (Fig. 1).

**Fig. 2:**
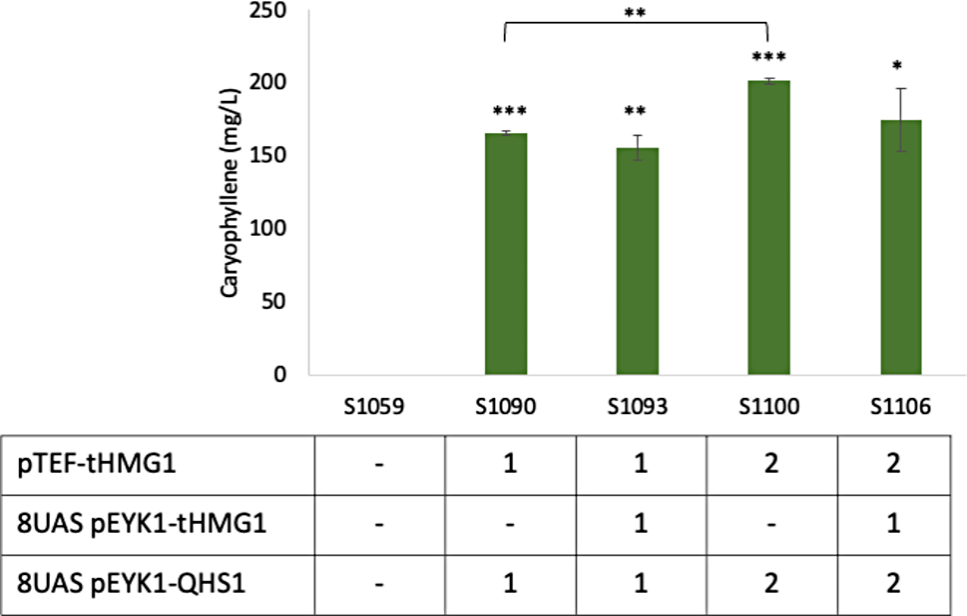
Production of β-caryophyllene in obese strains. The strains were grown in YPD4E1 (4% glucose and 1% erythritol) at 30 °C, 250 rpm for 96 h. The average and standard deviation were calculated from two biological replicates. Two-tailed T-test was performed and p values are indicated as asterisks in the graph (* *p* < 0.05, ** *p* < 0.01, ***p < 0.001)

The synthetic inducible promoter, 8UAS pEYK1 containing eight direct repeats of the upstream activating sequence B of *XPR2* gene (UAS B_XPR2_) and the core promoter of *EYK1* was used for gene expression in this study. This hybrid promoter already showed higher expression and stronger induction by inducer (erythritol or erythrulose) compared to the ones from the native promoter pEYK1 [21]. It was also described that the deletion of *EYK1* enhanced the expression of genes under pEYK1-based promoters by using erythritol as an inducer only not a carbon source for biomass [21, 22]. Thus, we deleted *EYK1* gene in the obese strain engineered for accumulating high amount of lipids and lacking the native *EYK1* gene to strongly express target genes (*QHS1* and/or *tHMG1*) under 8UAS pEYK1 promoter. The obese strain can be beneficial to increase the production of hydrophobic products like some terpenes as they can be stored in lipid bodies [23]. When the single copy of *QHS1* and *tHMG1* were expressed in the obese Δ*eyk1* strain (S1090), 165.4 mg/L of β-caryophyllene was produced (Fig. 2). Another copy of *QHS1* and *tHMG1* (S1100) produced higher β-caryophyllene by 1.22-fold. In order to see if we can boost the precursor by overexpressing *tHMG1*, we additionally expressed *tHMG1* under 8UAS pEYK1 promoter. In both strains, single and double copies of *QHS1* and *tHMG1* strains, the additional expression of *tHMG1* did not increase the production of β-caryophyllene (S1093 vs. S1090 and S1106 vs. S1100, respectively).

### Comparison of different genetic background for β-caryophyllene production

**Fig. 3:**
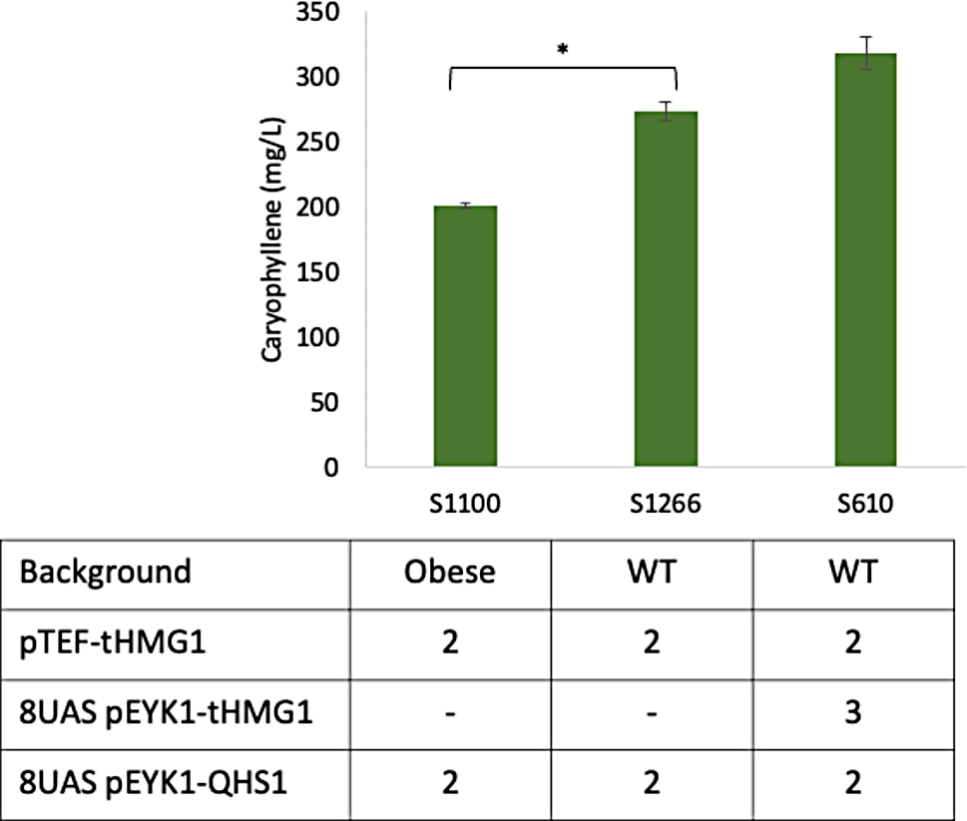
Comparison of β-caryophyllene production in different genetic backgrounds. The WT-based strains were grown in YPD1E3 (1% glucose and 3% erythritol) and the obese strain was grown in YPD4E1 (4% glucose and 1% erythritol) at 30 °C, 250 rpm for 96 h. The average and standard deviation were calculated from two biological replicates. Two-tailed T-test was performed and p values are indicated as asterisks in the graph (* *p* < 0.05, ** *p* < 0.01, ***p < 0.001)

The different genetic background often results in different production of target compound as shown in several references [15, 23]. As known for oleaginous yeast, *Y. lipolytica* has an advantage as a terpene producer due to its large intracellular acetyl-CoA pool. As acetyl-CoA is a common precursor of lipids and terpenes, we tried to verify if the acetyl-CoA is competitive for synthesizing two compounds thus affecting the synthesis of β-caryophyllene. The same gene expression cassettes of S1100 strain, 2 copies of *QHS1* and *tHMG1*, were introduced into the WT strain (*EYK1*+) to see if the production of β-caryophyllene is increased. The resulting strain, S1266 was cultivated in YNBD1E3 to adjust the carbon amount for growth compared to the condition for the obese Δ*eyk1* strains. The WT-based strain produced more β-caryophyllene by 1.36-fold than obese Δ*eyk1* strain with the same copy number of target genes. This might be due to the competition between lipid and terpene synthesis. In addition, the inhibition of the β-oxidation pathway in the obese strain might result in a decrease in acetyl-CoA accumulation from the degradation of lipids. This could lead to lower terpene production, as shown in other studies [24]. When we introduced the additional three cassettes of *tHMG1* to boost the precursor pool, the production of β-caryophyllene reached to 318.5 mg/L that is the highest titer from the flask cultivation (See Fig. 3).

### Optimization of substrates for β-caryophyllene production

**Fig. 4:**
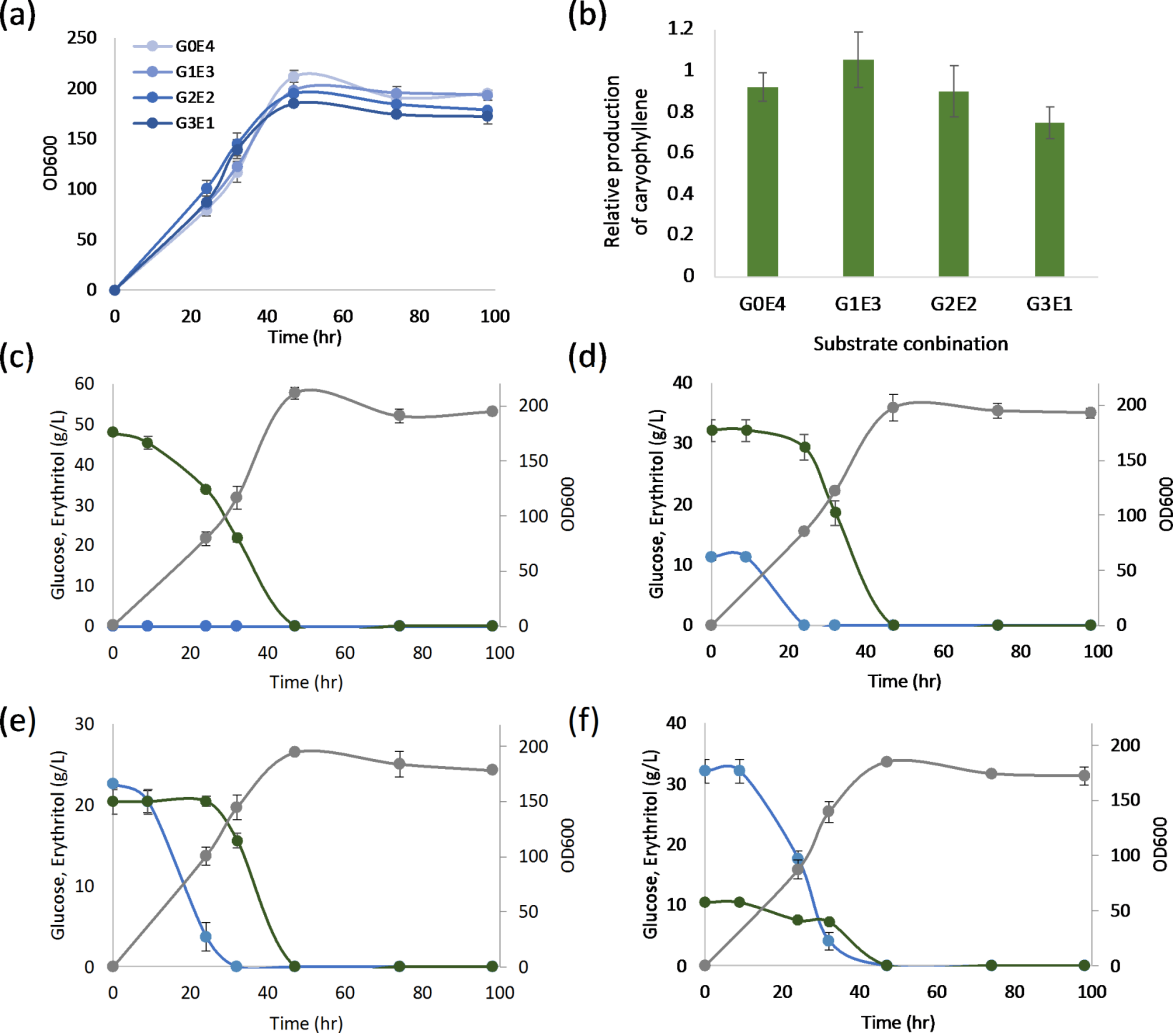
The effects of substrate concentration between glucose and erythritol. The S610 strains were grown in YP with different concentrations of glucose and erythritol at 30 °C, 250 rpm for 96 h. The average and standard deviation were calculated from duplicate biological repeat. (**a**) The growth of S610 strain with different combination of carbon sources. (**b**) The relative production of β-caryophyllene with different combination of carbon sources. (**c**-**f**) The consumption profile of carbon sources analyzed by HPLC. Glucose, blue; erythritol, green; OD, grey. The average and standard deviation were calculated from three biological replicates

Erythritol is used not only as a carbon source for the cell growth but also as an inducer for the gene expression when the pEYK1-based promoter is used in WT strain (*EYK1*+) [22]. To find the optimum concentration of erythritol for β-caryophyllene production, four different combinations of glucose and erythritol concentrations adjusted to the same carbon amount (G4E0, G3E1, G2E2, and G1E3) were tested with the best-performing strain, S610. The four conditions showed the similar growth patterns as described in Fig. 4 (a). All carbon sources were exhausted after 48 h of cultivation, and the erythritol consumption was started when the concentration of glucose is below around 10 g/L (Fig. 4 (c-f)). The best production of β-caryophyllene was obtained in G1E3 condition that is 1.14-fold higher than erythritol-only media (G0E4). Lower erythritol than G1E3 condition showed the less production of β-caryophyllene as described in Fig. 4 (b).

### Batch fermentation for β-caryophyllene production

**Fig. 5:**
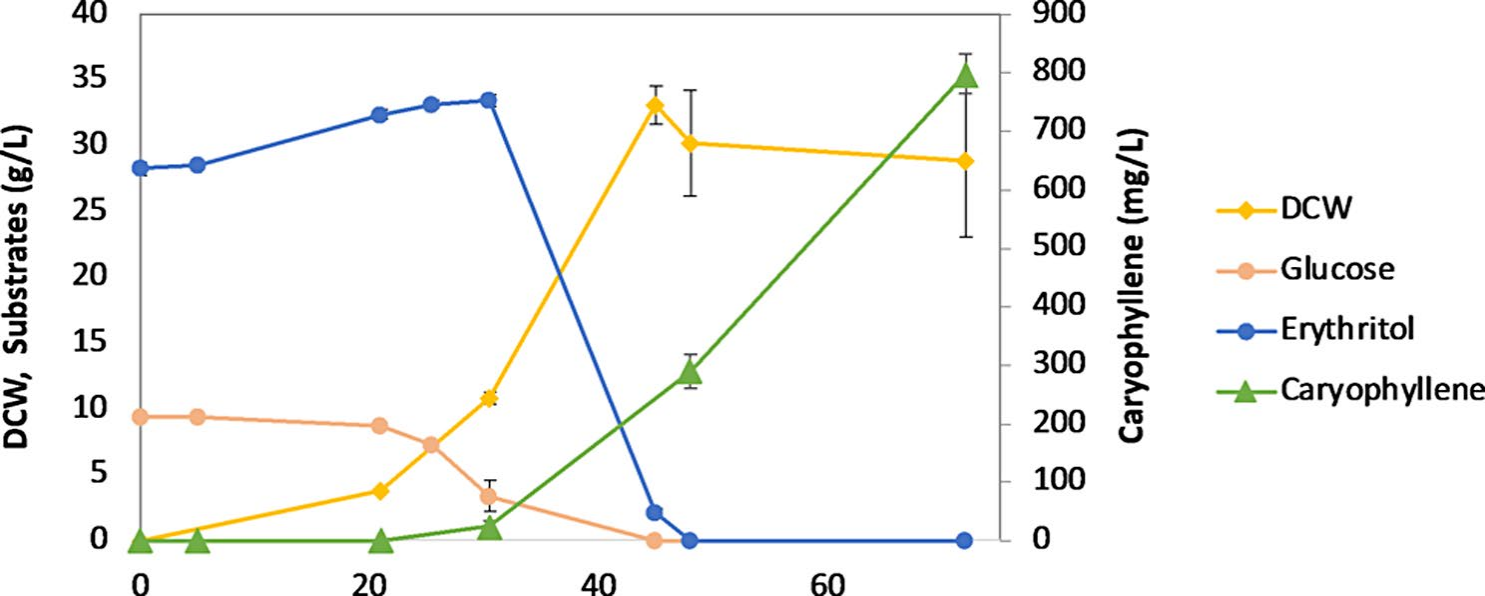
Batch fermentation of S610 for β-caryophyllene production. The average and standard deviation were calculated from two biological repeats

Batch fermentation was performed with the best-performing strain, S610, in YPD1E3 at 5 L-scale bioreactor to grow under controlled conditions and improve the production of β-caryophyllene (Fig. 5). The exponential growth phase lasted for 45 h with a specific growth rate and a final biomass of 0.37 h^− 1^ and 28.9 DCW g/L, respectively. Increase in erythritol at the beginning probably came from erythritol synthesis from glucose by the native pathway in *Y. lipolytica* [25, 26]. β-caryophyllene accumulation was started with the consumption of erythritol after 24 h and maintained until the end of batch fermentation, reaching 798.1 mg/L, the highest titer produced in *Y. lipolytica*. From this result, it is expected that fed-batch or chemostat fermentation, especially by optimizing inducer feeding strategy, could potentially increase the production of β-caryophyllene.

## Discussion

Elevating the expression of terpene synthase is pivotal for successful terpene production, and a simple yet very effective approach to enhance the production is by modulating gene expression levels. In this study, we aimed to apply a hybrid erythritol inducible promoter containing 8 tandem repeats of upstream activating sequences of *XPR2* (UAS) and core promoter of pEYK1 for stronger expression of β-caryophyllene synthase [20, 27]. For boosting precursor, *tHMG1* was expressed under the strong constitutive promoter pTEF as it is well known key enzyme for terpene biosynthesis. This combination of two enzymes led to 165.4 mg/L and 201.5 mg/L of β-caryophyllene production by single and double copies, respectively. It was observed that the augmentation of copy numbers for tHMG proved to be more effective on the expression under 8UAS pEYK1 compared to the pTEF promoter in erythritol-containing media (data not shown). With the utilization of erythritol-inducible promoter, optimizing induction strategies in fed-batch or chemostat fermentation could potentially lead to an improvement in the production of β-caryophyllene. Inducible promoters are favored as it dissociates the growth and production phase thus alleviating the metabolic load during protein expression [22]. As a hydrophilic inducer, erythritol has more advantages over other inducible promoters for *Y. lipolytica* such as pPOX2 and pLIP2 requiring hydrophobic inducers which is difficult to be dispersed in the culture medium [28]. For these reasons, erythritol-inducible promoter has been also selected in other studies (e.g. protein production such as lipase and human IgG in *Y. lipolytica*) [22, 29].

While the modulation of the expression of two key enzymes yielded β-caryophyllene production in this study, there are rooms for improvement through the application of various metabolic engineering strategies. Rewiring the MVA pathway to terpene synthesis has already shown successful improvement on various terpenes [1, 30, 31]. Identifying bottleneck steps in β-caryophyllene synthesis pathway by flux analysis and/or omics analysis would be instrumental in optimizing this strategy as it can vary based on the strains, target terpenes, and so on.

We compared two different genetic backgrounds for β-caryophyllene production, a wild-type and a high lipid-accumulating strain. Two copies of *QHS1* and *tHMG1* were expressed in both strains with glucose and erythritol. A WT strain performed better than an obese strain on β-caryophyllene production, reaching a 1.36-times higher titer. Previous studies on terpene production have shown controversial results regarding the relation between lipid accumulation and terpene synthesis [30]. The increase of the intracellular hydrophobic cell compartment by improving lipid content led to the increase in some terpenoids (β-carotene, lycopene, campesterol, and squalene) in *Y. lipolytica* [23, 32–34]. However, lipid synthesis can be competitive for terpene synthesis since the innate large pool of acetyl-CoA in *Y. lipolytica* is usually used for lipid synthesis. In addition, the obese strain was specifically engineered to accumulate high levels of lipids by inhibiting β-oxidation, a process that generates acetyl-CoA. Therefore, this engineering may not be conducive to terpene synthesis. Previous study inhibited lipid synthesis by supplementing cerulenin to improve amorphadiene production, resulting in 2.3 times higher titer [35]. Thus, achieving a delicate balance between lipid and terpene synthesis is crucial for constructing an efficient terpene producer.

Regarding competition of precursor between native metabolism and terpene synthesis, the compartmentalization can be a solution for improving β-caryophyllene production. Previous studies have shown successful compartmentalization of β-carotene, astaxanthin, and α-humulene synthesis in endoplasmic reticulum (ER), lipid droplets (LDs), or peroxisome in *Y. lipolytica* [31, 36, 37].

## Conclusions

β-Caryophyllene is of great interest in pharmaceutical, cosmetic, and chemical industries due to its therapeutic properties and potential as precursors of valuable chemicals. However, microbial production of β-caryophyllene, as an alternative to plant extraction or chemical synthesis, is still challenging regarding the production yields and costs. In this study, *Y. lipolytica* has been engineered to produce β-caryophyllene. The modulation of expression of key enzymes by inducible promoter and multi-copy integration resulted in an increase of β-caryophyllene production, reaching 318.5 mg/L from flask fermentation. By optimizing substrate concentration and batch fermentation under controlled condition, 798.1 mg/L of β-caryophyllene was produced. This study presents the potential of *Y. lipolytica* as a platform strain of terpene production.

## Electronic supplementary material

Below is the link to the electronic supplementary material.

Supplementary Material 1

## Data Availability

No datasets were generated or analysed during the current study.
