# Supplementary material for: Efficient biosynthesis of β-caryophyllene by engineered Yarrowia lipolytica
Source: Microb Cell Fact. 2025 Feb 6;24:38. doi: 10.1186/s12934-025-02660-w (PMC11800524; doi:10.1186/s12934-025-02660-w)
Supplement: Supplementary file 1 — Supplementary Material 1 [file 12934_2025_2660_MOESM1_ESM.docx]

**Supplementary Table 1. Microbial production of β-caryophyllene.**

|  | Origin of QHS1 | Production of β-caryophyllene | Substrate | Cultivation | Cultivation time | Reference |
| --- | --- | --- | --- | --- | --- | --- |
| *E. coli* | *Artemisia annua* | 106 mg/L  1.05 g/L | Acetic acid | Flask  Fed-batch fermentation | 60 hr after induction | Yang and Nie, 2016 |
| *E. coli* | *Artemisia annua* | 220 mg/L  1.52 g/L | Glucose | Flask  Fed-batch fermentation | 60 hr after induction | Yang et al. 2016 |
| *E. coli* | *Nicotiana tabacum* L | 100.3 mg/L  5.142 g/L | Glucose | Flask  Fed-batch fermentation | 72 hr | Cheng et al. 2022 |
| *S. cerevisiae* | *Artemisia annua* | 104.7 mg/L | Glucose | Test tube | 72 hr | Godara and Kao, 2021 |
| *S. cerevisiae* | *Artemisia annua* | 250.4 mg/L  2.949 g/L | Glucose | Flask  Fed-batch fermentation | 90 hr  140 hr | Li et al. 2021 |
| *Y. lipolytica* | *Artemisia annua* | 318.5 mg/L  798.1 mg/L | Glucose and  Erythritol | Flask  Batch fermentation | 120 hr | This study |

**Supplementary Table 2. Sequence of backbone plasmid and genes used in this study.**

| DCL-LEU2 ex | gcggccgcctgtcgggaaccgcgttcaggtggaacaggaccacctcccttgcacttcttggtatatcagtataggctgatgtattcatagtggggtttttcataataaatttactaacggcaggcaacattcactcggcttaaacgcaaaacggaccgtcttgatatcttctgacgcattgaccaccgagaaatagtgttagttaccgggtgagttattgttcttctacacaggcgacgcccatcgtctagagttgatgtactaactcagatttcactacctaccctatccctggtacgcacaaagcactttgctagatagagtcgagaattaccctgttatccctacataacttcgtatagcatacattatacgaagttattctgaattccgcctgagtcatcatttatttaccagttggccacaaacccttgacgatctcgtatgtcccctccgacatactcccggccggctgggtacgttcgatagcgctatcggcatcgacaaggtttgggtccctagccgataccgcactacctgagtcacaatcttcggaggtttagtcttccacatagcacgggcaaaagtgcgtatatatacaagagcgtttgccagccacagattttcactccacacaccacatcacacatacaaccacacacatccacaatggaacccgaaactaagaagaccaagactgactccaagaagattgttcttctcggcggcgacttctgtggccccgaggtgattgccgaggccgtcaaggtgctcaagtctgttgctgaggcctccggcaccgagtttgtgttcgaggaccgactcattggaggagctgccattgagaaggagggcgagcccatcaccgacgctactctcgacatctgccgaaaggctgactctattatgctcggtgctgtcggaggcgctgccaacaccgtatggaccactcccgacggacgaaccgacgtgcgacccgagcagggtctcctcaagctgcgaaaggacctgaacctgtacgccaacctgcgaccctgccagctgctgtcgcccaagctcgccgatctctcccccatccgaaacgttgagggcaccgacttcatcattgtccgagagctcgtcggaggtatctactttggagagcgaaaggaggatgacggatctggcgtcgcttccgacaccgagacctactccgttcctgaggttgagcgaattgcccgaatggccgccttcctggcccttcagcacaacccccctcttcccgtgtggtctcttgacaaggccaacgtgctggcctcctctcgactttggcgaaagactgtcacccgagtcctcaaggacgagttcccccagctggagctcaaccaccagctgatcgactcggccgccatgatcctcatcaagcagccctccaagatgaatggtatcatcatcaccaccaacatgtttggcgatatcatctccgacgaggcctccgtcatccccggttctctgggtctgctgccctccgcctctctggcttctctgcccgacaccaacgaggcgttcggtctgtacgagccctgtcacggatctgcccccgatctcggcaagcagaaggtcaaccccattgccaccattctgtctgccgccatgatgctcaagttctctcttaacatgaagcccgccggtgacgctgttgaggctgccgtcaaggagtccgtcgaggctggtatcactaccgccgatatcggaggctcttcctccacctccgaggtcggagacttgttgccaacaaggtcaaggagctgctcaagaaggagtaagtcgtttctacgacgcattgatggaaggagcaaactgacgcgcctgcgggttggtctaccggcaggatctgctagtgtataagactctataaaaagggccctgccctgctaatgaaatgatgatttataatttaccggtgtagcaaccttgactagaagaagcagattgggtgtgtttgtagtggaggacagtggtacgttttggaaacagtcttcttgaaagtgtcttgtctacagtatattcactcataacctcaatagccaagggtgtagtcggtttattaaaggaagggagttgtggctgatgtggatagatatctttaagctggcgactgcacccaacgagtgtggtggtagcttgttactgtatattcgaattcgtataacttcgtatagcaggagttatccgaagcgataattaccctgttatccctagaatcgatgatacgcgtccatggtgaggtgtctcacaagtgccgtgcagtcccgcccccacttgcttctctttgtgtgtagtgtacgtacattatcgagaccgttgttcccgcccacctcgatccggcatgctgaggtgtctcacaagtgccgtgcagtcccgcccccacttgcttctctttgtgtgtagtgtacgtacattatcgagaccgttgttcccgcccacctcgatccggcatgctgaggtgtctcacaagtgccgtgcagtcccgcccccacttgcttctctttgtgtgtagtgtacgtacattatcgagaccgttgttcccgcccacctcgatccggcatgctgaggtgtctcacaagtgccgtgcagtcccgcccccacttgcttctctttgtgtgtagtgtacgtacattatcgagaccgttgttcccgcccacctcgatccgacatgttgaggtgtctcacaagtgccgtgcagtcccgcccccacttgcttctctttgtgtgtagtgtacgtacattatcgagaccgttgttcccgcccacctcgatccggcatgctgaggtgtctcacaagtgccgtgcagtcccgcccccacttgcttctctttgtgtgtagtgtacgtacattatcgagaccgttgttcccgcccacctcgatccggcatgctgaggtgtctcacaagtgccgtgcagtcccgcccccacttgcttctctttgtgtgtagtgtacgtacattatcgagaccgttgttcccgcccacctcgatccggcatgctgaggtgtctcacaagtgccgtgcagtcccgcccccacttgcttctctttgtgtgtagtgtacgtacattatcgagaccgttgttcccgcccacctcgatccggcatgcactgatcacgggcaaaagtgcttcgatgcatctacttttctctatactgtacgtttcaatctggggaagcggaatcccaaaagggaaagccgccgcattaagctccacagccttgcataatccgatgacctgactagtgcggacaaagactattatttcgaggcaaggccaccacgtaccgcggtcccaaacttttgcaaagctgaaaacagcgtgggggtcaacgtggatcagaaagaggggcagatcagcttctataagaagctcctttccccacaattggcccacacgacacttctacacacttacacatctactggatctaagctttaagtcgactgggtggtatatatatatatatatatatatataactgtctagaaataaagagtatcatctttcaaagtgtctgtggtatctaagctatttatcactctttacaacttctacctcaactatctactttaataaatgaatatcgtttattctctatgattactgtatatgcgttcctctaagacaaatcgagagaccgggttggcggcgcatttgtgtcccaaaaaacagccccaattgccccaattgaccccaaattgacccagtagcggacccaaccccggcgagagcccccttcaccccacatatcaaacctcccccggttcccacacttgccgttaagggcgtagggtactgcagtctggaatctacgcttgttcagactttgtactagtttctttgtctggccatccgggtaacccatgccggacgcaaaatagactactgaaaatttttttgctttgtggttgggactttagccaagggtataaaagaccaccgtccccgaattacctttcctcttcttttctctctctccttgtcaactcacacccgaaggatcccacataatagcctagggctagggtgtctgtggtatctaagctatttatcactctttacaacttctacctcaactatctactttaataaatgaatatcgtttattctctatgattactgtatatgcgttcctctaagacaaatcgaattccatgtgtaacactcgctctggagagttagtcatccgacagggtaactctaatctcccaacaccttattaactctgcgtaactgtaactcttcttgccacgtcgatcttactcaattttcctgctcatcatctgctggattgttgtctatcgtctggctctaatacatttattgtttattgcccaaacaactttcattgcacgtaagtgaattgttttataacagcgttcgccaattgctgcgccatcgtcgtccggctgtcctaccgttaggggtagtgtgtctcacactaccgaggttactagagttgggaaagcgatactgcctcggacacaccacctgggtcttacgactgcagagagaatcggcgttacctctctcacaaagcccttcagtgcggccgcccggggtggcgaagaactccagcatgagatccccgcgctggaggatcatccagccggcgtcccggaaaacgattccgaagcccaacctttcatagaaggcggcggtggaatcgaaatctcgtgatggcaggttgggcgtcgcttggtcggtcatttcgaaccccagagtcccgctcagaagaactcgtcaagaaggcgatagaaggcgatgcgctgcgaatcgggagcggcgataccgtaaagcacgaggaagcggtcagcccattcgccgccaagctcttcagcaatatcacgggtagccaacgctatgtcctgatagcggtccgccacacccagccggccacagtcgatgaatccagaaaagcggccattttccaccatgatattcggcaagcaggcatcgccatgggtcacgacgagatcctcgccgtcgggcatgcgcgccttgagcctggcgaacagttcggctggcgcgagcccctgatgctcttcgtccagatcatcctgatcgacaagaccggcttccatccgagtacgtgctcgctcgatgcgatgtttcgcttggtggtcgaatgggcaggtagccggatcaagcgtatgcagccgccgcattgcatcagccatgatggatactttctcggcaggagcaaggtgagatgacaggagatcctgccccggcacttcgcccaatagcagccagtcccttcccgcttcagtgacaacgtcgagcacagctgcgcaaggaacgcccgtcgtggccagccacgatagccgcgctgcctcgtcctgcagttcattcagggcaccggacaggtcggtcttgacaaaaagaaccgggcgcccctgcgctgacagccggaacacggcggcatcagagcagccgattgtctgttgtgcccagtcatagccgaatagcctctccacccaagcggccggagaacctgcgtgcaatccatcttgttcaatcatgcgaaacgatcctcatcctgtctcttgatcagatcttgatcccctgcgccatcagatccttggcggcaagaaagccatccagtttactttgcagggcttcccaaccttaccagagggcgccccagctggcaattccggttcgcttgctgtccataaaaccgcccagtctagctatcgccatgtaagcccactgcaagctacctgctttctctttgcgcttgcgttttcccttgtccagatagcccagtagctgacattcatccggggtcagcaccgtttctgcggactggctttctacgtgttccgcttcctttagcagcccttgcgccctgagtgcttgcggcagcgtgaagctagcttatgcggtgtgaaataccgcacagatgcgtaaggagaaaataccgcatcaggcgctcttccgcttcctcgctcactgactcgctgcgctcggtcgttcggctgcggcgagcggtatcagctcactcaaaggcggtaatacggttatccacagaatcaggggataacgcaggaaagaacatgtgagcaaaaggccagcaaaaggccaggaaccgtaaaaaggccgcgttgctggcgtttttccataggctccgcccccctgacgagcatcacaaaaatcgacgctcaagtcagaggtggcgaaacccgacaggactataaagataccaggcgtttccccctggaagctccctcgtgcgctctcctgttccgaccctgccgcttaccggatacctgtccgcctttctcccttcgggaagcgtggcgctttctcatagctcacgctgtaggtatctcagttcggtgtaggtcgttcgctccaagctgggctgtgtgcacgaaccccccgttcagcccgaccgctgcgccttatccggtaactatcgtcttgagtccaacccggtaagacacgacttatcgccactggcagcagccactggtaacaggattagcagagcgaggtatgtaggcggtgctacagagttcttgaagtggtggcctaactacggctacactagaaggacagtatttggtatctgcgctctgctgaagccagttaccttcggaaaaagagttggtagctcttgatccggcaaacaaaccaccgctggtagcggtggtttttttgtttgcaagcagcagattacgcgcagaaaaaaaggatctcaagaagatcctttgatcttttctactgaacggtgatccccaccggaatt |
| --- | --- |
| JMP62-LEU2 ex-8UAS-EYK | aatcgatgatacgcgtccatggtgaggtgtctcacaagtgccgtgcagtcccgcccccacttgcttctctttgtgtgtagtgtacgtacattatcgagaccgttgttcccgcccacctcgatccggcatgctgaggtgtctcacaagtgccgtgcagtcccgcccccacttgcttctctttgtgtgtagtgtacgtacattatcgagaccgttgttcccgcccacctcgatccggcatgctgaggtgtctcacaagtgccgtgcagtcccgcccccacttgcttctctttgtgtgtagtgtacgtacattatcgagaccgttgttcccgcccacctcgatccggcatgctgaggtgtctcacaagtgccgtgcagtcccgcccccacttgcttctctttgtgtgtagtgtacgtacattatcgagaccgttgttcccgcccacctcgatccgacatgttgaggtgtctcacaagtgccgtgcagtcccgcccccacttgcttctctttgtgtgtagtgtacgtacattatcgagaccgttgttcccgcccacctcgatccggcatgctgaggtgtctcacaagtgccgtgcagtcccgcccccacttgcttctctttgtgtgtagtgtacgtacattatcgagaccgttgttcccgcccacctcgatccggcatgctgaggtgtctcacaagtgccgtgcagtcccgcccccacttgcttctctttgtgtgtagtgtacgtacattatcgagaccgttgttcccgcccacctcgatccggcatgctgaggtgtctcacaagtgccgtgcagtcccgcccccacttgcttctctttgtgtgtagtgtacgtacattatcgagaccgttgttcccgcccacctcgatccggcatgcactgatcacgggcaaaagtgcttcgatgcatctacttttctctatactgtacgtttcaatctggggaagcggaatcccaaaagggaaagccgccgcattaagctccacagccttgcataatccgatgacctgactagtgcggacaaagactattatttcgaggcaaggccaccacgtaccgcggtcccaaacttttgcaaagctgaaaacagcgtgggggtcaacgtggatcagaaagaggggcagatcagcttctataagaagctcctttccccacaattggcccacacgacacttctacacacttacacatctactggatcccacataatagcctagggtgtctgtggtatctaagctatttatcactctttacaacttctacctcaactatctactttaataaatgaatatcgtttattctctatgattactgtatatgcgttcctctaagacaaatcgaattccatgtgtaacactcgctctggagagttagtcatccgacagggtaactctaatctcccaacaccttattaactctgcgtaactgtaactcttcttgccacgtcgatcttactcaattttcctgctcatcatctgctggattgttgtctatcgtctggctctaatacatttattgtttattgcccaaacaactttcattgcacgtaagtgaattgttttataacagcgttcgccaattgctgcgccatcgtcgtccggctgtcctaccgttaggggtagtgtgtctcacactaccgaggttactagagttgggaaagcgatactgcctcggacacaccacctgggtcttacgactgcagagagaatcggcgttacctctctcacaaagcccttcagtgcggccgcccggggtggcgaagaactccagcatgagatccccgcgctggaggatcatccagccggcgtcccggaaaacgattccgaagcccaacctttcatagaaggcggcggtggaatcgaaatctcgtgatggcaggttgggcgtcgcttggtcggtcatttcgaaccccagagtcccgctcagaagaactcgtcaagaaggcgatagaaggcgatgcgctgcgaatcgggagcggcgataccgtaaagcacgaggaagcggtcagcccattcgccgccaagctcttcagcaatatcacgggtagccaacgctatgtcctgatagcggtccgccacacccagccggccacagtcgatgaatccagaaaagcggccattttccaccatgatattcggcaagcaggcatcgccatgggtcacgacgagatcctcgccgtcgggcatgcgcgccttgagcctggcgaacagttcggctggcgcgagcccctgatgctcttcgtccagatcatcctgatcgacaagaccggcttccatccgagtacgtgctcgctcgatgcgatgtttcgcttggtggtcgaatgggcaggtagccggatcaagcgtatgcagccgccgcattgcatcagccatgatggatactttctcggcaggagcaaggtgagatgacaggagatcctgccccggcacttcgcccaatagcagccagtcccttcccgcttcagtgacaacgtcgagcacagctgcgcaaggaacgcccgtcgtggccagccacgatagccgcgctgcctcgtcctgcagttcattcagggcaccggacaggtcggtcttgacaaaaagaaccgggcgcccctgcgctgacagccggaacacggcggcatcagagcagccgattgtctgttgtgcccagtcatagccgaatagcctctccacccaagcggccggagaacctgcgtgcaatccatcttgttcaatcatgcgaaacgatcctcatcctgtctcttgatcagatcttgatcccctgcgccatcagatccttggcggcaagaaagccatccagtttactttgcagggcttcccaaccttaccagagggcgccccagctggcaattccggttcgcttgctgtccataaaaccgcccagtctagctatcgccatgtaagcccactgcaagctacctgctttctctttgcgcttgcgttttcccttgtccagatagcccagtagctgacattcatccggggtcagcaccgtttctgcggactggctttctacgtgttccgcttcctttagcagcccttgcgccctgagtgcttgcggcagcgtgaagctagcttatgcggtgtgaaataccgcacagatgcgtaaggagaaaataccgcatcaggcgctcttccgcttcctcgctcactgactcgctgcgctcggtcgttcggctgcggcgagcggtatcagctcactcaaaggcggtaatacggttatccacagaatcaggggataacgcaggaaagaacatgtgagcaaaaggccagcaaaaggccaggaaccgtaaaaaggccgcgttgctggcgtttttccataggctccgcccccctgacgagcatcacaaaaatcgacgctcaagtcagaggtggcgaaacccgacaggactataaagataccaggcgtttccccctggaagctccctcgtgcgctctcctgttccgaccctgccgcttaccggatacctgtccgcctttctcccttcgggaagcgtggcgctttctcatagctcacgctgtaggtatctcagttcggtgtaggtcgttcgctccaagctgggctgtgtgcacgaaccccccgttcagcccgaccgctgcgccttatccggtaactatcgtcttgagtccaacccggtaagacacgacttatcgccactggcagcagccactggtaacaggattagcagagcgaggtatgtaggcggtgctacagagttcttgaagtggtggcctaactacggctacactagaaggacagtatttggtatctgcgctctgctgaagccagttaccttcggaaaaagagttggtagctcttgatccggcaaacaaaccaccgctggtagcggtggtttttttgtttgcaagcagcagattacgcgcagaaaaaaaggatctcaagaagatcctttgatcttttctactgaacggtgatccccaccggaattgcggccgcctgtcgggaaccgcgttcaggtggaacaggaccacctcccttgcacttcttggtatatcagtataggctgatgtattcatagtggggtttttcataataaatttactaacggcaggcaacattcactcggcttaaacgcaaaacggaccgtcttgatatcttctgacgcattgaccaccgagaaatagtgttagttaccgggtgagttattgttcttctacacaggcgacgcccatcgtctagagttgatgtactaactcagatttcactacctaccctatccctggtacgcacaaagcactttgctagatagagtcgagaattaccctgttatccctacataacttcgtatagcatacattatacgaagttattctgaattccgcctgagtcatcatttatttaccagttggccacaaacccttgacgatctcgtatgtcccctccgacatactcccggccggctgggtacgttcgatagcgctatcggcatcgacaaggtttgggtccctagccgataccgcactacctgagtcacaatcttcggaggtttagtcttccacatagcacgggcaaaagtgcgtatatatacaagagcgtttgccagccacagattttcactccacacaccacatcacacatacaaccacacacatccacaatggaacccgaaactaagaagaccaagactgactccaagaagattgttcttctcggcggcgacttctgtggccccgaggtgattgccgaggccgtcaaggtgctcaagtctgttgctgaggcctccggcaccgagtttgtgttcgaggaccgactcattggaggagctgccattgagaaggagggcgagcccatcaccgacgctactctcgacatctgccgaaaggctgactctattatgctcggtgctgtcggaggcgctgccaacaccgtatggaccactcccgacggacgaaccgacgtgcgacccgagcagggtctcctcaagctgcgaaaggacctgaacctgtacgccaacctgcgaccctgccagctgctgtcgcccaagctcgccgatctctcccccatccgaaacgttgagggcaccgacttcatcattgtccgagagctcgtcggaggtatctactttggagagcgaaaggaggatgacggatctggcgtcgcttccgacaccgagacctactccgttcctgaggttgagcgaattgcccgaatggccgccttcctggcccttcagcacaacccccctcttcccgtgtggtctcttgacaaggccaacgtgctggcctcctctcgactttggcgaaagactgtcacccgagtcctcaaggacgagttcccccagctggagctcaaccaccagctgatcgactcggccgccatgatcctcatcaagcagccctccaagatgaatggtatcatcatcaccaccaacatgtttggcgatatcatctccgacgaggcctccgtcatccccggttctctgggtctgctgccctccgcctctctggcttctctgcccgacaccaacgaggcgttcggtctgtacgagccctgtcacggatctgcccccgatctcggcaagcagaaggtcaaccccattgccaccattctgtctgccgccatgatgctcaagttctctcttaacatgaagcccgccggtgacgctgttgaggctgccgtcaaggagtccgtcgaggctggtatcactaccgccgatatcggaggctcttcctccacctccgaggtcggagacttgttgccaacaaggtcaaggagctgctcaagaaggagtaagtcgtttctacgacgcattgatggaaggagcaaactgacgcgcctgcgggttggtctaccggcaggatctgctagtgtataagactctataaaaagggccctgccctgctaatgaaatgatgatttataatttaccggtgtagcaaccttgactagaagaagcagattgggtgtgtttgtagtggaggacagtggtacgttttggaaacagtcttcttgaaagtgtcttgtctacagtatattcactcataacctcaatagccaagggtgtagtcggtttattaaaggaagggagttgtggctgatgtggatagatatctttaagctggcgactgcacccaacgagtgtggtggtagcttgttactgtatattcgaattcgtataacttcgtatagcaggagttatccgaagcgataattaccctgttatccctag |
| *tHMG* | atggccgctgtcctgaccaacaagaccgtgatctctggttctaaggtgaagtctctgtcctccgcccagtcttcttcctccggtccctcctcttcctccgaggaggacgactcccgagacatcgagtccctggacaagaagatccgacccctggaggagctggaggccctgctttcctccggtaacaccaagcagctgaagaacaaggaggtggccgccctggtcatccacggcaagctgcctctgtacgccctggagaagaagctgggtgacaccacccgagccgtggccgttcgacgaaaggccctgtccattctggccgaggcccccgttctggcctccgaccgacttccttacaagaactacgactacgaccgagtgttcggcgcctgctgtgagaacgtgatcggctacatgcccctgcccgtcggtgtgatcggccctcttgtcattgacggtacttcttaccacattcccatggccaccaccgagggttgtctggtggcctccgccatgcgaggctgcaaggctattaacgccggcggcggtgccaccaccgtgctgaccaaggacggcatgacccgaggccccgtggtccgattccccactctgaagcgatccggtgcctgcaagatttggctggactccgaggagggtcagaacgccatcaagaaggccttcaactccacctctcgattcgcccgactgcagcacatccagacctgtctggccggcgacctgctgttcatgcgattccgaaccaccaccggcgacgccatgggtatgaacatgatttccaagggtgtcgagtactccctgaagcagatggtcgaggagtacggctgggaggacatggaggtcgtgtctgtctctggtaactactgcaccgacaagaagcccgccgccattaactggattgagggtcgaggtaagtctgtcgtcgccgaggccaccatccccggagatgtcgtccgaaaggtcctgaagtccgacgtctctgccctggtcgagctgaacatcgccaagaacctggtgggctccgccatggccggttctgtgggaggattcaacgcccacgccgccaacctggtgaccgctgttttcctggccctgggccaggaccccgctcagaacgttgagtcctctaactgcatcaccctgatgaaggaggtggacggcgacctgcgaatttctgtgtccatgccctccatcgaggtgggcaccattggtggcggtactgtcctggagccccagggtgctatgctggacctgctgggcgtccgaggtccccatgctaccgctcctggcaccaacgcccgacagctggctcgaatcgtcgcctgcgccgtgctggctggagagctttccctgtgcgccgccctggctgctggtcatctggtccagtcccacatgacccacaaccgaaagcccgccgagcccaccaagcccaacaacctggacgccaccgacattaaccgactgaaggacggctctgtgacctgcattaagtcctaa |
| *QHS1* | atgtctgtgaaggaggagaaggtcatccgacccatcgtgcacttccccccctccgtgtgggccgaccagttcctgatcttcgacgacaagcaggccgagcaggctaacgtcgagcaggtggtgaacgagctgcgagaggacgtgcgaaaggacctggtctcttctctggacgtccagaccgagcacaccaacctgctgaagctgattgacgccatccagcgactgggcatcgcctaccacttcgaggaggagatcgagcaggccctgcagcacatctatgacacctacggtgacgactggaagggccgatctccctctctgtggttccgaatcctgcgacagcagggcttctacgtctcttgcgacattttcaagaactacaagaaggaggacggctctttcaaggagtccctgaccaacgacgtggagggtctgctggagctgtacgaggccacctacctgcgagtccagggtgagggcgtgctggacgacgccctggtcttcacccgaacctgtctggagaagattgccaaggacctggtgcacaccaaccccaccctgtccacctacatccaggaggccctgaagcagcccctgcacaagcgactgacccgactggaggccctgcgatacattcccatgtacgagcagcaggcctctcacaacgagtccctgctgaagctcgccaagctgggtttcaacctgctgcagtccctgcaccgaaaggagctgtccgaggtctcccgatggtggaagggtctggacgtgcccaacaacctgccctacgcccgagaccgaatggtcgagtgttacttctgggccctgggtgtctacttcgagcccaagtactctcaggcccgaattttcctggccaaggtcatttccctggccaccgtgctggacgatacctacgacgcctacggcacctacgaggagctgaagattttcaccgaggccattcagcgatggtctatcacctgtatcgacatgctgcccgagtacctgaagctgctgtaccagggcgtgctggatatctatattgagatggaggagattatgggtaaagagggcaaggcccaccacctgtcctacgccaaggagtccatgaaggagttcatccgatcctacatgatggaggccaagtgggctaacgagggctacgtccccaccgccgaggagcacatgtctgtggccttcgtctcttctggctactctatgctggccaccacctgtttcgtcggtatgggcgacattgtgaccgacgaggccttcaagtgggccctgaccaagccccccattattaaggcctcctgcgccatcgcccgactgatggacgacatccactcccagaaggaggagaaggagcgaattcacgtggcctcctctgtggagtcttacatgaagcagtacgacgtgaccgaggagcacgtgctgaaggtgttcaacaagaagatcgaggacgcctggaaggacatcacccgagagtctctggtgcgaaaggatatccccatgcccctgatgatgcgagtcatcaacctggcccaggtcatggacgtcctgtacaagcacaaggacggcttcaccaacgtcggtgaggagctgaaggaccacattaagtctctgctggtccaccccatccccatctaa |

**Supplementary Table 3. Primer list used in this study.**

| Primer name | Sequence 5’ − 3’ |
| --- | --- |
| pTEF-Start-Fw | GCTTTGTGGTTGGGACTTTAGCCAAGGG |
| pTEF-internal-Fw | CCATGCCGGACGCAAAATAGACTAC |
| pEYK-internal-Fw | GTACGTTTCAATCTGGGGAAGCGG |
| tHMGR1-Sc-Rev | CCTTCAGTCGGTTAATGTCGGTGGCGTCCAGG |
| QHS1-Fw | ATGTCTGTGAAGGAGGAGAAGGTCATCC |
| QHS1-Rev | TTAGATGGGGATGGGGTGGACCAGCAGAG |
| QHS1-internal-Fw | GATGTAGGTGGACAGGGTGGGG |
